# Diphtheria Outbreak in Amerindian Communities, Wonken, Venezuela, 2016–2017

**DOI:** 10.3201/eid2407.171712

**Published:** 2018-07

**Authors:** Adriana Lodeiro-Colatosti, Udo Reischl, Thomas Holzmann, Carlos E. Hernández-Pereira, Alejandro Rísquez, Alberto E. Paniz-Mondolfi

**Affiliations:** Instituto de Salud Pública del Estado Bolívar, Bolívar, Venezuela (A. Lodeiro-Colatosti);; Infectious Diseases Research Incubator, Barquisimeto, Venezuela (A. Lodeiro-Colatosti, C.E. Hernández-Pereira);; Zoonosis and Emerging Pathogens Regional Collaborative Network, Barquisimeto (A. Lodeiro-Colatosti, C.E. Hernández-Pereira);; University Hospital of Regensburg, Regensburg, Germany (U. Reischl, T. Holzmann);; Universidad Centroccidental Lisandro Alvarado, Barquisimeto (C.E. Hernández-Pereira);; Universidad Central de Venezuela, Caracas, Venezuela (A. Rísquez);; Instituto de Investigaciones Biomédicas IDB, Cabudare, Venezuela (A.E. Paniz-Mondolfi);; Instituto Venezolano de los Seguros Sociales, Caracas (A.E. Paniz-Mondolfi)

**Keywords:** *Corynebacterium diphtheriae*, diphtheria, outbreak, Venezuela, Amerindians, Great Savannah, Savannah Plateau, case-fatality rate, mining, epidemiology, clinical presentation, indigenous population, bacteria, respiratory infections, vaccination, Wonken

## Abstract

In February 2017, a diphtheria outbreak occurred among Amerindians of the Pemón ethnic group in Wonken, Venezuela. A field investigation revealed ≈10 cases; clinical presentation did not include cutaneous or neurologic signs or symptoms. To prevent future outbreaks in Venezuela, Amerindian communities need better access to vaccination and healthcare.

Diphtheria is a contagious acute bacterial infection caused by toxin-producing, gram-positive *Corynebacterium diphtheriae* and other *Corynebacteria* ssp., such as *Corynebacterium ulcerans* (*1*,*2*). Humans are a known reservoir, but bacteria can also be isolated from horses and cats. Transmission occurs primarily through contact with airborne respiratory secretions or exudation from infected skin lesions (*3*–*5*). The incidence of diphtheria in the Western Hemisphere has decreased dramatically over the past few decades, although the disease has remained endemic in some developing countries around the globe. Diphtheria was eradicated in Venezuela 25 years ago; the last reported case occurred in 1992 (*6*).

However, in November 2016, the International Health Regulations National Focal Point of Venezuela updated the Pan American Health Organization and World Health Organization about diphtheria in the country, reporting that 16 of 24 federal agencies had reported 183 suspected cases of the disease during September–November 2016 (*6*). During weeks 1–49 of 2017, suspected and confirmed diphtheria cases were reported in 4 countries in the Americas: Brazil (4 cases), the Dominican Republic (3 cases), Haiti (152 probable cases), and Venezuela (227 cases) (*7*).

## The Study

In February 2017, a cluster of ≈10 cases of an illness characterized by swollen neck occurring in 7 children and 3 adults (including 2 deaths) was reported in 3 Amerindian communities (Urimpatá [5.128429°N, –61.380956°E]; Damasko [5.127997°N, –61.504152°E]; Atanao [5.128429°N, –61.380956°E]) of the Great Savannah in Bolivar, Venezuela (Table). These settlements, which are part of the greater Weiyekupotá community, are home to the seminomadic populations of the Pemón aboriginals, who migrate for long periods to perform agricultural, hunting, fishing, and mining activities, with regular return visits to their home villages. Reaching these isolated communities can only be achieved by river navigation or small aircraft. Access to healthcare for this population is limited (≈2-day walk to closest hospital); according to reports from the Ministry of Health, the estimated diphtheria vaccination coverage rates during the first half of 2016 were <24%. This cluster of diphtheria cases prompted an epidemiologic investigation in the affected communities.

**Table T1:** Demographics and clinical characteristics of 10 Amerindians with suspected diphtheria cases, Wonken, Venezuela, 2017*

Case-patient no.	Age, y/sex	Location†	Signs and symptoms	Duration	Treatment	Outcome
1	31/M, returning miner	Urimpatá	Hyperthermia; dysphagia; odynophagia; dysphonia; gray adherent membranes; massive cervical lymphadenopathy	9 d	Azithromycin (500 mg, 2×/d for 10 d), 7-d cycle ampicillin/sulbactam, penicillin G benzathine (1.2 million units, IM, 1 dose), adult Td to contacts	Survived
2	4/F, household contact of case-patient 1	Urimpatá	Dysphagia; odynophagia; hemoptysis; fever; gray adherent membrane formation; cervical lymphadenopathy	7 d	Azithromycin (10 mg/kg,1×/d for 7 d), cefotaxime/clarithromycin at admission, Tdap vaccination	Survived, admitted to reference hospital
3	9/F, household contact of case-patient 1	Urimpatá	Abrupt onset of odynophagia; barking cough; dysphonia; stridor and gray adherent pseudomembranes covering tonsils, uvula, and pharynx	7 d	Azithromycin (10 mg/kg, 1×/d, 7 d), penicillin G benzathine (0.6 million units, IM, 1 dose), Tdap vaccination	Survived
4	14/F	Atanao	Fever; dysphonia; dysphagia; odynophagia	≈1 wk	No data	Died
5	4/M	Atanao	Dysphagia; odynophagia; dysphonia; hyporexia	≈1 wk	No data	Died
6	9/F	Urimpatá	Odynophagia; barking cough; dysphonia; stridor and gray pseudomembrane covering tonsils, uvula, and pharynx	≈1 wk	Azithromycin (10 mg/kg, 1×/d, 7 d), penicillin G benzathine (0.6 million units, IM, 1 dose), Tdap vaccination	Survived
7	9/F	Damasko	Dysphagia; odynophagia; dysphonia; fever; gray pseudomembrane covering tonsils, uvula, and pharynx	≈1 wk	Azithromycin (10 mg/kg, 1×/d for 7 d), penicillin G benzathine (0.6 million units, IM, 1 dose), Tdap vaccination	Survived
8	13/F	Damasko	Odynophagia; fever; small grayish membranes admixed with vesicles covering pharynx	≈1 wk	Azithromycin (10 mg/kg, 1×/d for 7 d), penicillin G benzathine (0.6 million units, IM, 1 dose), Tdap vaccination	Survived
9	Unknown	Atanao, in transit to Vista Alegre community	Reported as signs and symptoms suggestive of diphtheria	Unknown	No data	Unknown
10	Unknown	Atanao, in transit to Vista Alegre community	Reported as signs and symptoms suggestive of diphtheria	Unknown	No data	Unknown

*None of the case-patients were previously immunized or received diphtheria antitoxin as treatment. No case-patients had cutaneous or neurologic signs or symptoms. IM, intramuscular; Td, tetanus-diphtheria; Tdap, tetanus-diphtheria-acellular pertussis. †Location coordinates: Urimpatá (5.128429°N, −61.380956°E); Atanao (5.128429°N, −61.380956°E); and Damasko (5.127997°N, −61.504152°E).

**Figure 1:**
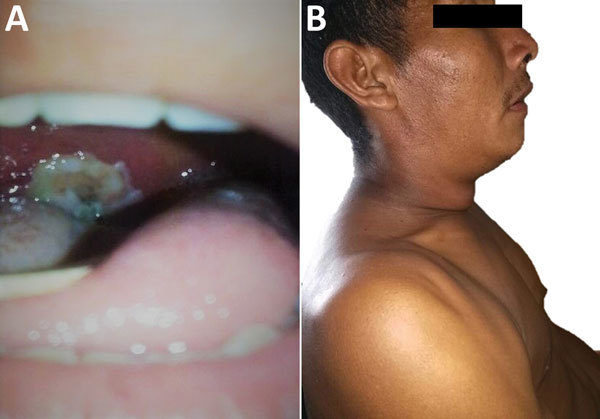
Physical characteristics of 31-year-old Amerindian male index case-patient with diphtheria, Wonken, Venezuela, 2017. A) Firmly adherent gray-white pseudomembrane in pharynx. B) Typical bull-like neck swelling with massive cervical adenopathies.

In Urimpatá, a 31-year-old Amerindian man (case-patient 1) who had recently returned home from a gold mining camp in Apoipó (4.744573°N, –61.477692°E) and 2 members of his household, his 4-year-old daughter (case-patient 2) and 9-year-old niece (case-patient 3), sought treatment for symptoms they had been experiencing for over a week. All 3 exhibited classic signs of diphtheria (Figure 1; Table) and did not have cutaneous lesions or neurologic signs or symptoms.

Pharyngeal samples from the index case-patient were collected on swabs and applied to glass slides, which were submitted for real-time PCR testing, as previously described (*8*). Compared with collecting the sample by scraping the dried sample from the glass slide, collecting the sample by rubbing the slide with a moist swab (wetted with phosphate-buffered saline) led to ≈100-fold higher yields of DNA in subsequent PCR assays. Samples were positive for *C. diphtheriae* toxin gene (*tox*) by real-time reverse transcription PCR; we observed cycle thresholds of ≈30 and the characteristic melting temperature by LightCycler hybridization probe (Sigma-Aldrich, St. Louis, MO, USA) melting curve analysis.

Persons with suspected diphtheria were given penicillin G benzathine and azithromycin [Table T1](Table). Because erythromycin and penicillin G procaine were not available and to broaden antimicrobial coverage, we additionally gave case-patient 1 a 7-day course of ampicillin/sulbactam and case-patient 2 cefotaxime/clarithromycin. Case-patient 2 was transferred to the nearest hospital for further assistance. None of the case-patients identified in this outbreak were given diphtheria antitoxin because of supply shortages nationwide. A few days before case-patients 1–3 sought treatment, 2 deaths were reported in Atanao in persons exhibiting the same symptoms: a 14-year-old girl (died in the community) and 4-year-old boy (transferred to Boa Vista, Brazil, and died later) (Table). Our team could not reach the rest of the case-patients with suspected diphtheria in distant mines and villages, but local personnel registered cases in adult miners in Atanao. None of these case-patients had been previously immunized. All 41 Amerindians examined by the investigation team and their contacts from 3 different villages received toxoid immunization.

Conclusions

Although diphtheria is declining or has been eliminated from many countries because of high and widespread immunization coverage, the disease remains endemic to some developing countries, especially in regions under extreme poverty and low vaccine coverage (*3*). Over the past 4 years, Venezuela has faced a sharp reduction in oil revenue and undergone economic and political developments that have led to high inflation, impoverishment, and scarcity of basic resources largely affecting the public health infrastructure, resulting in long-term shortages of essential medicines and medical supplies, including vaccines for universal immunization programs and the immunization of specific risk groups against specific diseases (*9*). In addition, job shortages have pushed many locals into the practice of informal economy, food speculation, and, particularly, illegal gold mining.

**Figure 2:**
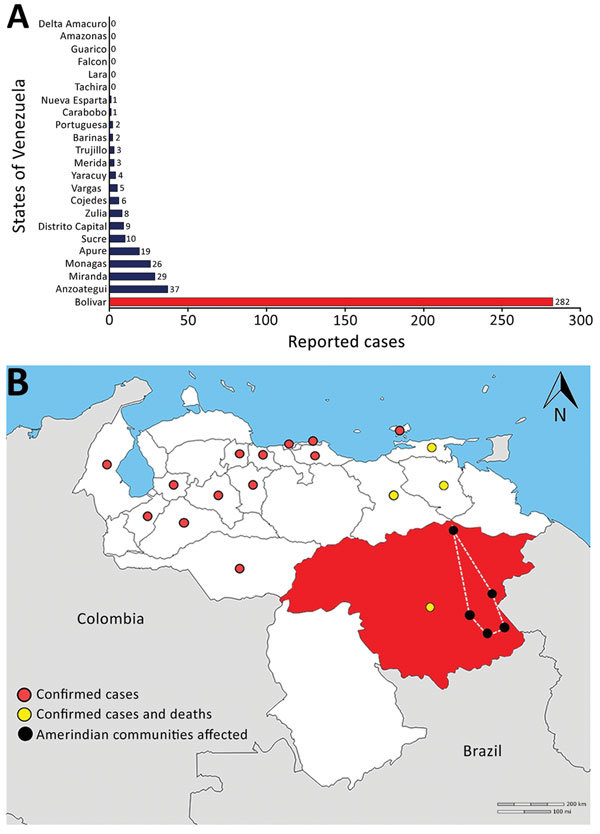
Suspected and confirmed diphtheria cases and deaths, by state, Venezuela, 2016–2017. The highest number of cases occurred in the state where Amerindians reside (Bolivar, red). A) Number of suspected cases of diphtheria reported from week 28 of 2016 through week 24 of 2017, by state. B) Location of confirmed cases and deaths, Venezuela, 2017. The affected Amerindian communities reside in the area within the dotted line. Map obtained from d-maps (http://d-maps.com/carte.php?num_car=4080&lang=es).

The state of Bolivar is the largest federal entity in the country and the richest in mineral deposits. Legal and illegal mining activity is ongoing and rapidly growing, especially since the government announced the uncontrolled opening of the mining arch of the Orinoco River in 2011. This situation has led to an unprecedented increase in vectorborne disease transmission in these areas (*8*). From week 1 in 2016 through week 48 in 2017, a total of 609 suspected cases were reported in Venezuela, 227 of which were laboratory confirmed, with a case-fatality rate (CFR) of 15.5% (*7*). As of week 24 in 2017, a total of 282 (63%) cases were reported from Bolivar (Figure 2, panels A, B), with most occurring in the highly populated municipalities of Heres and Sifontes (*10*). However, to the best of our knowledge, diphtheria cases among the isolated Amerindian communities of the Savannah Plateau we examined has not been reported elsewhere.

Diphtheria is primarily controlled by vaccination and ensuring optimal herd immunity through high immunization coverage (*3*). The occurrence of diphtheria outbreaks reflects inadequate vaccination coverage. This outbreak was probably the consequence of the reintroduction of previously eradicated diseases by infected migrants traveling through mining districts and low vaccination rates.

Although calculated as 15.5%, the CFR of this epidemic cannot be accurately estimated because of the geographic isolation and elusive nature of most Amerindian communities. However, the CFR is expected to be higher because of the low vaccination rates and complete absence of effective diphtheria treatments in most of the region.

This outbreak highlights 2 issues: the unknown epidemiologic effect of diphtheria on isolated, immunologically naive Amerindian tribes in Venezuela and the difficulty of diagnosing diphtheria when clinicians are unfamiliar with the disease, tribe members have limited access to healthcare, and doctors lack treatment and laboratory facilities. Of note, the diagnosis of 1 diphtheria case was made by using pharyngeal samples applied to glass slides that were later processed by molecular methods; the enhanced DNA detection seen by using wet swabs is a valuable observation, potentially making diagnosis more accessible for resource-poor communities.

Reports of diphtheria affecting other aboriginal communities in Venezuela, such as the Kariña population (Gran Kashaama, Guanipa Plateau, Anzoategui), indicate that further investigation is necessary to elucidate the true extent of diphtheria. The public health challenge of improving the provision of preventive services and access to medical care for the isolated and underserved communities in Bolivar is needed to prevent future diphtheria outbreaks.

9. Fraser B. Data reveal state of Venezuelan health system. Lancet. 2017;389:2095. 10.1016/S0140-6736(17)31435-628560998
